# Increased extracellular fluid is associated with white matter fiber degeneration in CADASIL: in vivo evidence from diffusion magnetic resonance imaging

**DOI:** 10.1186/s12987-021-00264-1

**Published:** 2021-06-30

**Authors:** Xinfeng Yu, Xinzhen Yin, Hui Hong, Shuyue Wang, Yeerfan Jiaerken, Fan Zhang, Ofer Pasternak, Ruiting Zhang, Linglin Yang, Min Lou, Minming Zhang, Peiyu Huang

**Affiliations:** 1 Department of Radiology, The 2nd Affiliated Hospital, Zhejiang University School of Medicine, No.88 Jiefang Road, Shangcheng District, Hangzhou, 310009 China; 2 Department of Neurology, The 2nd Affiliated Hospital, Zhejiang University School of Medicine, Hangzhou, China; 3 Department of Radiology, Brigham and Women’s Hospital, Harvard Medical School, Boston, MA USA; 4 Department of Psychiatry, Brigham and Women’s Hospital, Harvard Medical School, Boston, MA USA; 5 Department of Psychiatry, The 2nd Affiliated Hospital, Zhejiang University School of Medicine, Hangzhou, China

**Keywords:** White matter, Extracellular fluid, Cerebral small vessel diseases, CADASIL, MRI, Diffusion-weighted imaging

## Abstract

**Background:**

White matter hyperintensities (WMHs) are one of the hallmarks of cerebral small vessel disease (CSVD), but the pathological mechanisms underlying WMHs remain unclear. Recent studies suggest that extracellular fluid (ECF) is increased in brain regions with WMHs. It has been hypothesized that ECF accumulation may have detrimental effects on white matter microstructure. To test this hypothesis, we used cerebral autosomal-dominant arteriopathy with subcortical infarcts and leukoencephalopathy (CADASIL) as a unique CSVD model to investigate the relationships between ECF and fiber microstructural changes in WMHs.

**Methods:**

Thirty-eight CADASIL patients underwent 3.0 T MRI with multi-model sequences. Parameters of free water (FW) and apparent fiber density (AFD) obtained from diffusion-weighted imaging (b = 0 and 1000 s/mm^2^) were respectively used to quantify the ECF and fiber density. WMHs were split into four subregions with four levels of FW using quartiles (FWq1 to FWq4) for each participant. We analyzed the relationships between FW and AFD in each subregion of WMHs. Additionally, we tested whether FW of WMHs were associated with other accompanied CSVD imaging markers including lacunes and microbleeds.

**Results:**

We found an inverse correlation between FW and AFD in WMHs. Subregions of WMHs with high-level of FW (FWq3 and FWq4) were accompanied with decreased AFD and with changes in FW-corrected diffusion tensor imaging parameters. Furthermore, FW was also independently associated with lacunes and microbleeds.

**Conclusions:**

Our study demonstrated that increased ECF was associated with WM degeneration and the occurrence of lacunes and microbleeds, providing important new insights into the role of ECF in CADASIL pathology. Improving ECF drainage might become a therapeutic strategy in future.

**Supplementary Information:**

The online version contains supplementary material available at 10.1186/s12987-021-00264-1.

## Introduction

White matter hyperintensities (WMHs) on MRI scans are one of the hallmarks of cerebral small vessel disease (CSVD), which are associated with cognitive impairment, dementia, stroke and even death [1–3]. However, the pathological mechanisms leading to WMHs remain unclear. WMHs are the earliest and most frequent observed imaging feature in cerebral autosomal-dominant arteriopathy with subcortical infarcts and leukoencephalopathy (CADASIL) [4] which is a monogenic CSVD caused by Notch 3 gene mutations [5]. Unlike sporadic CSVD, the occurrence and progression of WMHs are less dependent on cardiovascular risk factors and age in CADASIL [6]. Therefore, CADASIL could serve as a unique model for investigating the pathological mechanisms of WMHs.

Accumulating studies of CADASIL provide the evidence that WMHs are strongly related to increased extracellular fluid (ECF). For example, a neuropathological study has demonstrated that WMHs in the anterior temporal lobe of CADASIL reflect fluid accumulation in enlarged perivascular spaces (PVS), together with the loss of WM [7]. A recent study of CADASIL patients reveals that changes in diffusion MRI signals within WMHs are mainly driven by increased extracellular free water [8]. Furthermore, ECF are differentially distributed in the WMHs, with higher ECF in specific regions of WMHs such as anterior temporal lobe [9]. However, the association between increased ECF and white matter (WM) microstructural changes in WMHs has not yet been determined. It has been argued that increased ECF would result in a build-up of substances toxic (e.g., plasma proteins) to the WM microstructure [10, 11]. Although diffusion tensor imaging (DTI) is a well-established method for quantifying microstructural WM alterations in vivo, the model fitting would be largely influenced by the extracellular water and intravoxel crossing fibers which happen to be seen in WMHs [12].

Recent diffusion MRI methods applying novel post-processing approaches can be used to achieve more direct in vivo characterization of both intra- and extracellular properties of WM tissue. For example, free water (FW) imaging using bi-tensor model could potentially separate the diffusion signals from extracellular FW and tissue compartment [13]. Apparent fiber density (AFD) imaging using constrained spherical deconvolution (CSD) allows the resolution of crossing fibers in voxels containing multiple fiber orientations, and it could estimates the fraction of space occupied by a fiber bundle [14].

In this study, in a cohort of patients with CADASIL, we characterized the ECF (indexed by FW) and fiber density (indexed by AFD) in WMHs using the two advanced diffusion approaches. We hypothesized that increased ECF would be associated with fiber microstructural changes in WMHs. Considering the heterogeneity of ECF distribution in WMHs, we classified FW in individual WMHs into four levels using quartiles and used lesion probability map (LPM) to understand the distribution of different levels of ECF in WMHs. We analyzed the relationships between different levels of ECF and microstructural diffusion metrics (including AFD and FW-corrected DTI metrics). Given that WMHs commonly accompany lacunes and microbleeds, we further explored the correlations between ECF in WMHs with both lacunes and microbleeds.

## Methods

### Participants

The study was approved by the Medical Ethics Committee of the Second Affiliated Hospital, Zhejiang University School of Medicine and written informed consent was obtained from all subjects. We enrolled 38 patients with CADASIL. The diagnosis of CADASIL was confirmed by genetic testing with a pathogenic mutation in the Notch 3 gene which has been described in our previous study [15]. The following clinical variables were documented for each subject: age, gender, the history of hypertension, diabetes mellitus, hyperlipidemia, and smoking. In addition, the presence/absence of symptoms including transient ischemic attack/stroke, headache, psychiatric and cognitive impairments were also recorded. The disease duration was defined as the length of time between initial symptom onset and brain MRI examination.

### Data acquisition

All patients were examined using a 3.0 T whole body-scanner (Discovery MR750, GE Healthcare, Milwaukee, WI) equipped with an 8-channel phased array head coil. The details of MR sequence are shown in Additional file 1: Methods.

### Image processing

**Fig. 1:**
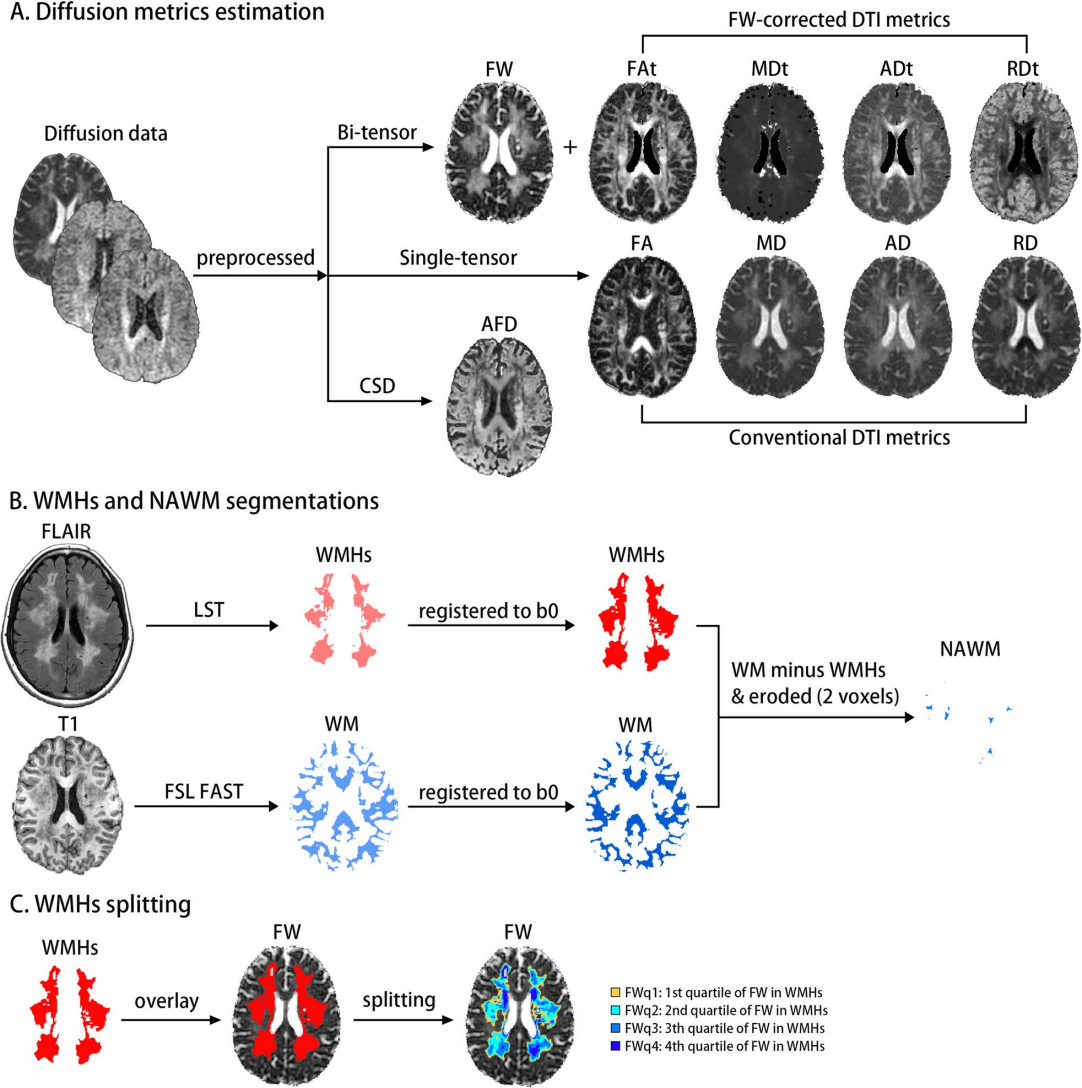
Overview of the image processing pipeline for each subject. **A** The single-shell diffusion data (b = 0, 1000 s/mm^2^) were preprocessed and then were separately fitted into bi-tensor, single tensor and CSD models. The diffusion parameters including FW, FW-corrected DTI metrics (FAt, MDt, ADt, RDt) and conventional DTI metrics (FA, MD, AD, RD) were estimated. **B** WMHs mask was created by FLAIR image using the toolbox of LST in SPM12 and WM mask was generated from T1 image using FSL FAST. Both masks of WMHs and WM were co-registered into the individual b = 0 s/mm^2^ (b0) image using ANTs. Then, NAWM was made by subtracting the WMHs mask from the WM mask, and it was subsequently eroded by two voxels to avoid partial volume effects. **C** The mask of WMHs was split into four sub-masks with using FW quartiles. *CSD*  constrained spherical deconvolution, *FW* free water; *DTI* diffusion tensor imaging, *FAt* tissue compartment FA, *MDt* tissue compartment MD; *ADt* tissue compartment AD, *RDt* tissue compartment RD, *FA* fractional anisotropy, *MD* mean diffusivity, *AD* axial diffusivity; *RD* radial diffusivity, *AFD* apparent fiber density; *WMHs* white matter hyperintensities, *FLAIR* T2 flu-id-attenuated inversion recovery; LST = Lesion Segmentation Toolbox; *SPM* Statistical Parametric Mapping, *WM* white matter, *FSL* FMRIB Software Library, *FAST* FMRIB's Automated Segmentation Tool, *ANTs* Advanced Normalization Tools, *NAWM* normal appearing white matter

Overview of the image processing pipeline for each subject is shown in Fig. 1.

#### Diffusion preprocessing

The preprocessing steps of diffusion data included denoising, eddy-current and echo-planar imaging (EPI) distortion correction and motion correction, gibbs ringing removal, bias-field correction. In addition to EPI distortion, the preprocessing steps were processed using a combination of commands in the MRtrix3 package, FMRIB Software Library (FSL) and Advanced Normalization Tools (ANTs). The EPI distortion was corrected using the bdp correction algorithm implemented in the BrainSuite software package (https://brainsuite.org).

#### Free water estimation

FW analysis has been described in detail elsewhere [8, 13, 16]. Briefly, the preprocessed diffusion data was fitted with a bi-tensor model within each voxel: the first one models FW diffusion, defining as an isotropic diffusion, and the second one models the tissue compartment. The isotropic compartment models extracellular FW which is characterized by freely and not hindered or restricted diffusion. The fraction of FW content per voxel provided a native FW map and varied between 0 and 1. FW-corrected DTI parameters were further calculated from the tissue compartment’s tensor, namely fractional anisotropy (FAt), mean diffusivity (MDt), axial diffusivity (ADt) and radial diffusivity (RDt). Besides, the conventional DTI parameters including FA, MD, AD and RD were estimated from the preprocessed diffusion data using linear least squares method [13]. Both FW-corrected and conventional DTI parameters were computed using Matlab R2018b (MathWorks, Natick, MA).

#### Apparent fiber density estimation

The preprocessed diffusion data were up-sampled, resulting in 1 mm^3^ isotropic voxels. Subsequently, the 3-tissue response functions including WM, gray matter (GM), and cerebrospinal fluid (CSF) were directly estimated from the diffusion data themselves for each subject using a robust and fully automated unsupervised method [17]. Then, the response functions obtained from all subjects for each tissue type were averaged. The WM fiber orientation distributions (FODs) for each participant were computed using the group averaged response function via single-shell 3-tissue CSD (SS3T-CSD) in MRtrix3Tissue [18]. Next, the WM FODs were further performed by bias fields correction and global intensity normalization [19]. Finally, the voxel-based AFD map was obtained from the integral of WM FODs [14].

#### Segmentations of WMHs and NAWM

WMHs were segmented using the FLAIR image by the lesion prediction algorithm implemented in the Lesion Segmentation Toolbox version 3.0.0 (www.statistical-modelling.de/lst.html) in Statistical Parametric Mapping Version 12. This algorithm consists of a binary classifier in the form of a logistic regression model trained on the data of 53 multiple sclerosis patients with severe lesion patterns [20]. Next, we used a threshold of 0.5 to obtain the binary mask for WMHs [21]. The mask of WMHs for each subject was visually checked and further manually corrected for false positives and negatives by an experienced neuroradiologist (X.F.Y) using ITK-SNAP software (www.itksnap.org). Then, WMHs mask was co-registered with the b = 0 s/mm^2^ (b0) image using the transformation matrix of registration from FLAIR image to b0 image with default parameters using ANTs. The final mask of WMHs (b0 space) was further checked by the same neuroradiologist (X.F.Y).

WM mask was created by the skull-stripped T1 image using FMRIB's Automated Segmentation Tool. Then, WM mask was co-registered with the b0 image using the transformation matrix of registration from T1 image to b0 image with default parameters using ANTs. Normal appearing white matter (NAWM) mask for each subject was created by subtracting the mask of WMHs from the mask of WM (both masks at the b0 space), and it was subsequently eroded by two voxels in three dimensions to ensure voxels within the final NAWM mask were not contaminated by partial volume effects. The neuroradiologist (X.F.Y) visually checked the NAWM mask to manually exclude the undesired voxels such as those from WMHs, lacunes, microbleeds and CSF.

To classify extracellular FW at different levels in WMHs, each subject’s WMHs mask was split into four sub-masks (FWq1 to FWq4) using FW quartile values within their own WMHs. FWq1 represents the subregions containing lowest 25% of FW values in WMHs, while FWq4 represents the subregions containing highest 25% of FW values in WMHs.

At last, the mean values of diffusion metrics for each subject were extracted from individual masks of WMHs, NAWM as well as each sub-mask of WMHs.

### Creating lesion probability map for FW

LPM was used to assess the spatial distribution of FW at each level as previously described [22, 23]. Firstly, T1 images (b0 space) were normalized to the Montreal Neurological Institute (MNI152) standard space (1 mm^3^), and the corresponding matrices were applied to each sub-mask of WMHs with nearest-neighbor interpolation using ANTs. All registrations were checked visually to exclude alignment failures. Then, LPM for each level of FW was generated by averaging the corresponding sub-mask of WMHs using fslmaths tool (part of FSL). For each LPM, the resulting voxel intensity indicates how frequently the voxel in question is within a lesion across all patients (i.e., the probability of that voxel being lesional).

### Estimation of intracranial volume

Intracranial volumes (ICV) were estimated using T1 image by automatic image processing pipeline of FreeSurfer version 6.0 (https://surfer.nmr.mgh.harvard.edu). The total WMHs volume and four subregions of WMHs volumes were all corrected by ICV.

### Evaluations of lacunes and microbleeds

The presence of lacunes and microbleeds were evaluated by two radiologists (Y.X.F and H.H). Lacunes were defined as 3- to 15-mm cavitated lesions (CSF-like signals on T1 and FLAIR images) of presumed ischemic origin and distinct from dilated perivascular spaces [24]. Microbleeds were defined as small (diameter ≤ 10 mm) and rounded hypointense lesions on SWI images and microbleeds mimics, such as vessels, calcification, partial volumes and air/bone interfaces were carefully excluded [24]. The inter-rater agreements for the presence of lacunes and microbleeds between two raters were determined by the Cohen’s kappa.

### Statistics

Comparisons of diffusion metrics between WMHs and NAWM were performed by paired-t test, and among the four WMHs subregions were analyzed using one-way multivariate analysis of variance (MANOVA) which were followed by post hoc tests with Bonferroni correction. The effect sizes were estimated using generalized eta squared (eta^2^[g]). Because the conventional DTI metrics were contaminated by FW, we did not include them in the correlation analysis. We performed the correlations between FW, AFD with FW-corrected DTI metrics using Pearson’s correlation. The correlations were further analyzed using partial correlation, after adjusting age, gender and corrected WMHs volume. The correlation coefficients were considered significant at p < 0.05 following Bonferroni adjustment for multiple comparisons. We additionally analyzed the relationships between duration of disease with FW and AFD using partial correlation, after adjusting age, gender and corrected WMH volumes. We used logistics regression model to identify the relationships between FW with lacunes and microbleeds. The odds ratio (OR) and its 95% confidence intervals (CI) were estimated. Change-in-estimate approach was used to select variables in a stepwise fashion according to the magnitude of the differences between adjusted and unadjusted effect estimates [25]. A change in the estimated measure of association of 10% or more would be evidence that confounding was present [26]. All statistical analyses were performed in R (version 4.0.3) and SPSS (version 26.0).

## Results

Table 1 summarizes the demographic, clinical characteristics and neuroimaging features of CADASIL patients.

**Table 1:** Demographic, clinical, and neuroimaging characteristics of CADASIL patients

	CADASIL (n = 38)
Age, mean (SD), y	49.7 (6.5)
Gender (female), n (%)	27 (71.1)
Duration of disease, median (IQR), y	2 (0.8—5)
Hypertension, n (%)	6 (15.8)
Diabetes mellitus, n (%) Hyperlipidemia, n (%) Smoking, n (%) TIA/Stroke, n (%) Headache, n (%) Cognitive impairment, n (%) Psychiatric impairment, n (%) WMHs volume, mean (SD), ml Corrected WMHs volume by ICV, mean (SD) With lacunes, n (%) With microbleeds, n (%)	3 (7.9) 3 (7.9) 3 (7.9) 18 (47.4) 22 (57.9) 14 (36.8) 17 (44.7) 81.1 (40.0) 0.057 (0.027) 27 (71.1) 21 (55.3)

*CADASIL* Cerebral autosomal dominant arteriopathy with subcortical infarcts and leukoencephalopathy, *SD* standard deviation, *IQR* interquartile range, *TIA * transient ischemic attack, *WMHs* white matter hyperintensities, *ICV* intracranial volumes

### Abnormal diffusion metrics in WMHs

**Fig. 2:**
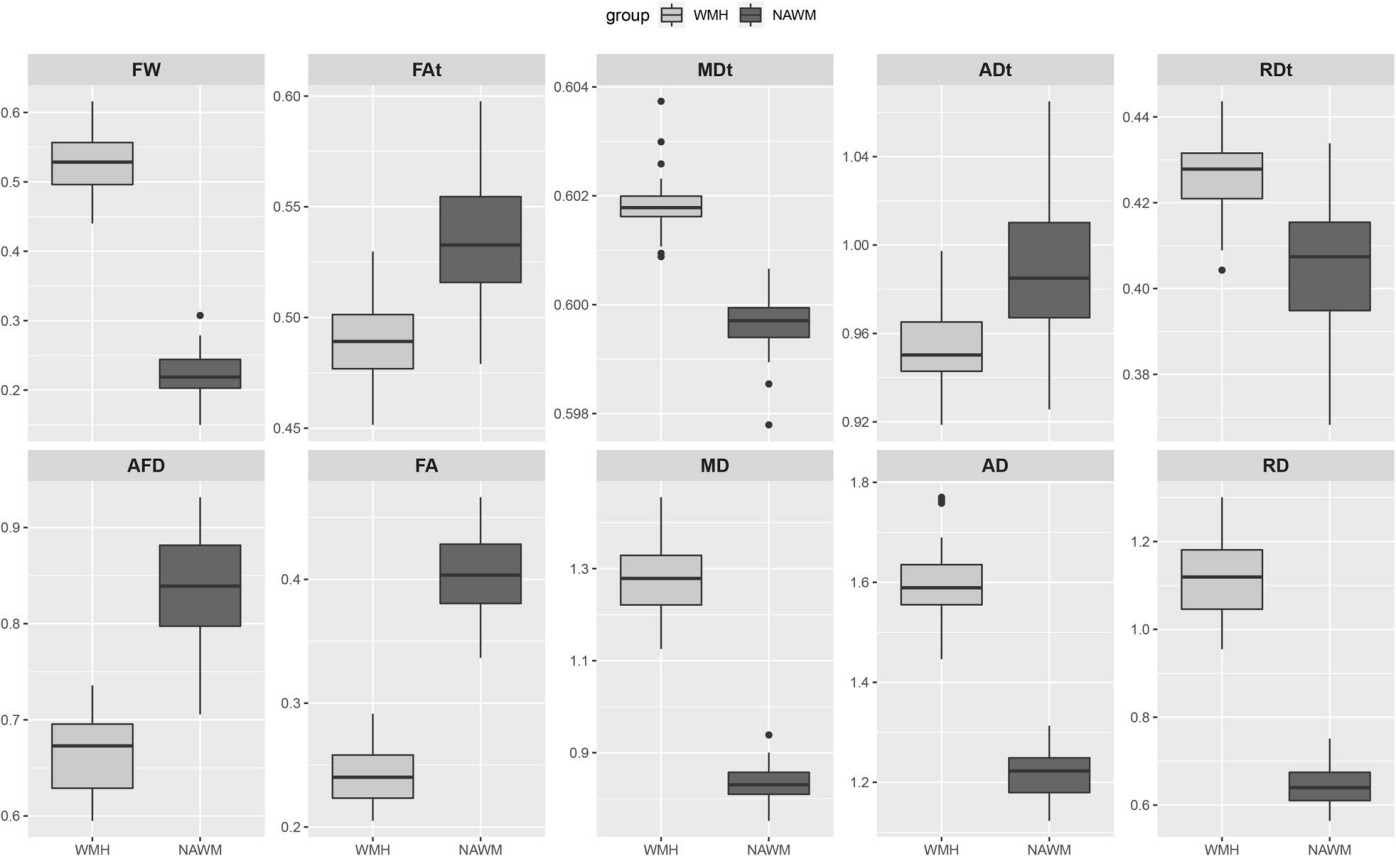
Abnormal diffusion metrics in WMHs: comparisons between WMHs and NAWM. Significant differences were found in all diffusion metrics including FW, AFD, FAt, MDt, ADt, RDt, FA, MD, AD and RD (all *p* < 0.001). The MDt, ADt, RDt, MD, AD and RD values are in units of × 10^−3^ mm^2^/s. WMHs = white matter hyperintensities; *NAWM* normal appearing white matter, *FW* free water, *AFD* apparent fiber density, *FAt* tissue compartment FA, *MDt* tissue compartment MD, *ADt* tissue compartment AD, *RDt* tissue compartment RD, *FA * fractional anisotropy, *MD* mean diffusivity, *AD* axial diffusivity, *RD* radial diffusivity

Compared with NAWM, FW increased and AFD decreased in WMHs. FAt and ADt were lower in WMHs than in NAWM, while MDt and RDt was higher. For conventional DTI metrics, we found the similar changes in FA, MD and RD, except in AD which showed a change in the opposite direction. The results are shown in Fig. 2.

### Correlations between diffusion metrics in WMHs

**Fig. 3:**
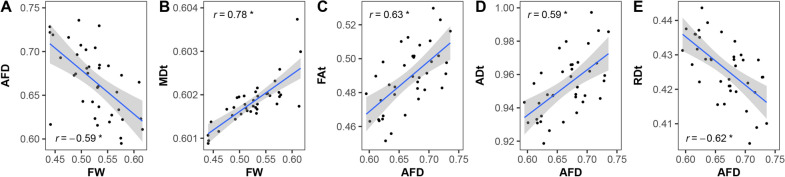
Correlations between FW, AFD with FW-corrected DTI metrics in WMHs. **A**, **B** FW correlated with AFD and MDt (both *p* < 0.001), and **C**, **E** AFD correlated with FAt, ADt and RDt (all *p* < 0.001). The MDt, ADt and RDt values are in units of × 10^−3^ mm^2^/s. *Indicates *p* < 0.0001. *FW* free water, *AFD* apparent fiber density, *DTI* diffusion tensor imaging, *WMHs* white matter hyperintensities, *MDt* tissue compartment mean diffusivity, *Fat* tissue compartment fractional anisotropy, *ADt* tissue compartment axial diffusivity, *RDt* tissue compartment radial diffusivity

FW was only significantly correlated with AFD and MDt, and AFD was significantly correlated with FAt, ADt and RDt (Fig. 3). The correlations were slightly stronger after adjusting age, gender and corrected WMHs volumes (Additional file 1: Table S1).

### Lesion probability maps for different levels of FW

**Fig. 4:**
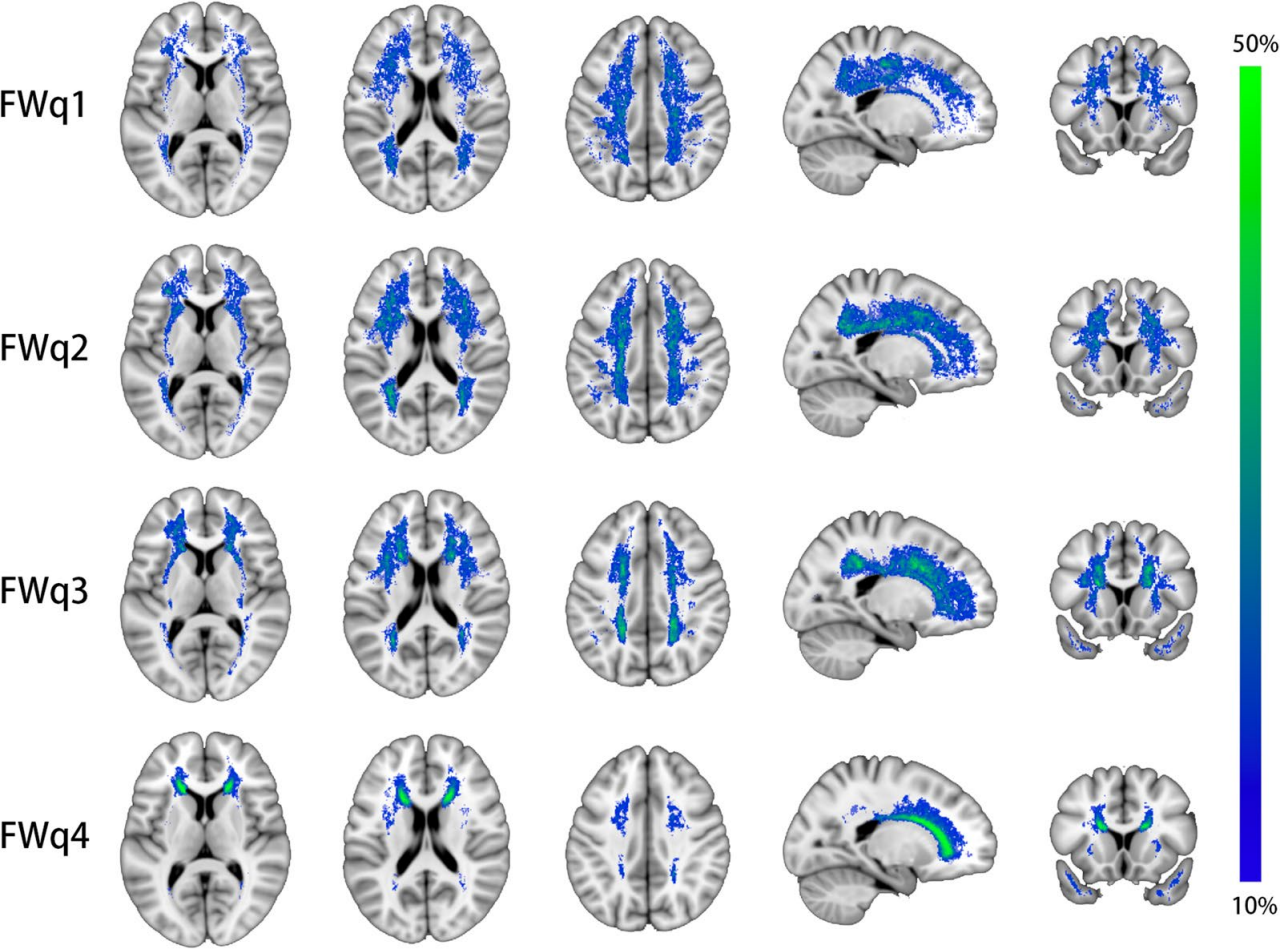
Distribution pattern of FW in four subregions of WMHs (FWq1-4). The highest frequency of lesion voxels was identified in the anterior horn of the lateral ventricle in FWq4 (peak probability = 0.55, voxel size = 182 mm^3^). *FW* free water, *WMHs* white matter hyperintensities, *FWq1-4* four subregions of WMHs split by FW quartiles

Figure 4 displays LPMs for FWq1 to FWq4. The lesion distribution of FWq1 appeared to demonstrate a diffusely distributed pattern, mainly in the deep WM. With the increased FW, the more frequent involvement of areas tended to move towards the lateral ventricle (i.e., periventricular predilection). Involvement of anterior temporal lobe was also more frequently seen in subregions with high-level FW (FWq3 and FWq4).

### Abnormal diffusion metrics in subregions with different levels of FW

**Fig. 5:**
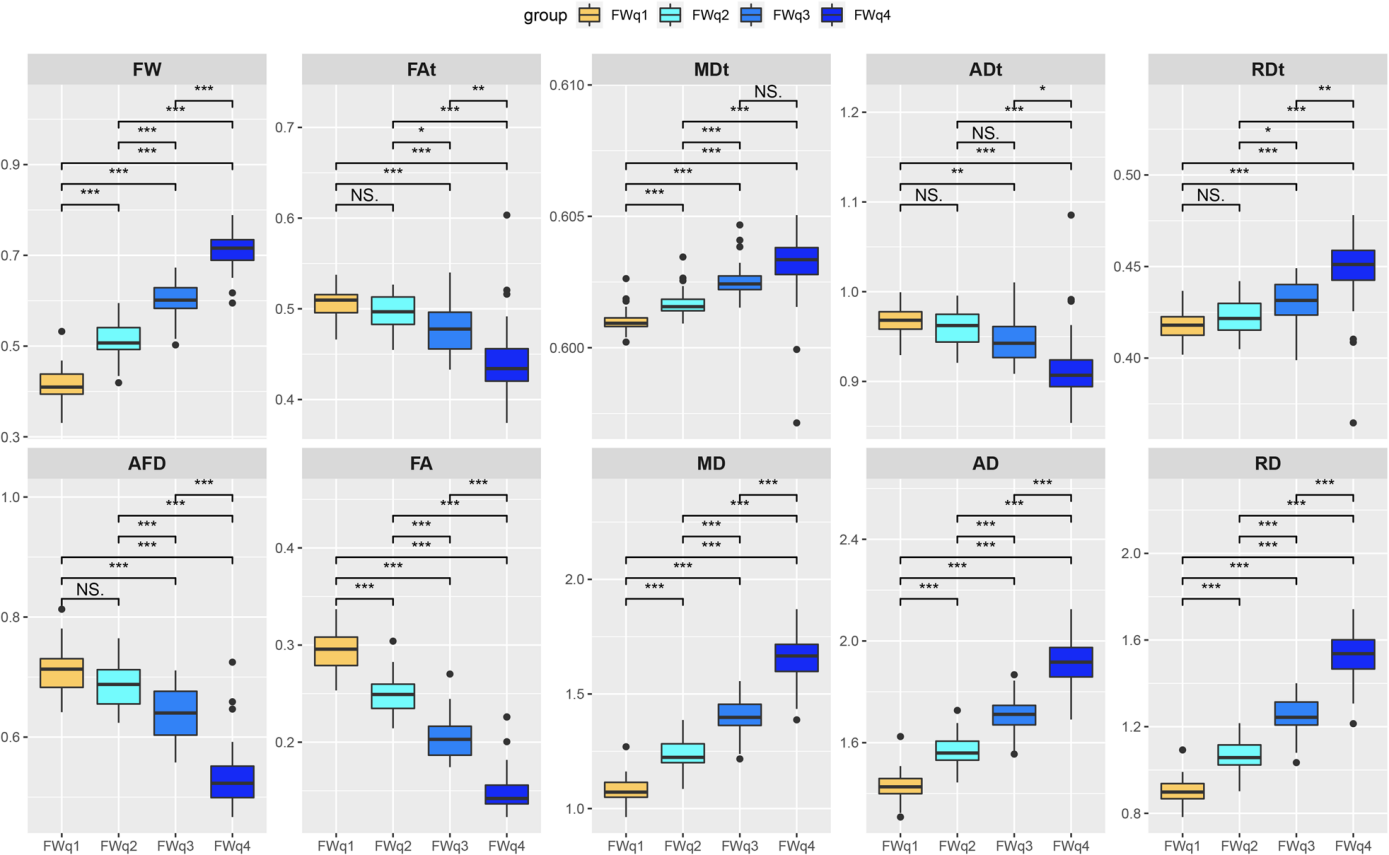
Abnormal diffusion metrics in WMHs: comparisons among four subregions of WMHs (FWq1-4). Alterations in in all diffusion metrics including FW, AFD, FAt, MDt, ADt, RDt, FA, MD, AD, RD (all *p* < 0.001) were found. The pairwise comparisons between two subregions were performed by post hoc analysis for each diffusion metric. In addition to AFD, FAt, ADt and RDt between FWq1 and FWq2 (*p* = 0.036, *p* = 0.214, *p* = 0.554 and *p* = 0.263, respectively), MDt between FWq3 and FWq4 (*p* = 0.06) and ADt between FWq2 and FWq3 (p = 0.025), the comparisons between two subregions for each diffusion metrics reached significance by Bonferroni adjustment. The MDt, ADt, RDt, MD, AD and RD values are in units of × 10^−3^ mm^2^/s. *Indicates Bonferroni corrected *p* < 0.05; **Indicates Bonferroni corrected p < 0.01; ***Indicates Bonferroni corrected *p* < 0.001; NS. Indicates not significant. *WMHs* white matter hyperintensities, *FW* free water, *AFD* apparent fiber density, *FAt* tissue compartment fractional anisotropy, *MDt* tissue compartment mean diffusivity; ADt = tissue compartment axial diffusivity, *RDt* tissue compartment radial diffusivity; FA = fractional anisotropy, *MD* mean diffusivity, *AD* axial diffusivity; *RD* radial diffusivity; FWq1-4 = four subregions of WMHs split by FW quartiles

Figure 5 shows comparisons of all diffusion metrics among the four subregions of WMHs. Because each diffusion metric was significantly higher or lower in FWq1 than those in NAWM (Additional file 1: Figure S1), NAWM was not included in the MANOVA analysis. From FWq1 to FWq4, a decreasing gradient was found in AFD (F = 120.46, eta2[g] = 0.71), FAt (F = 37.80, eta2[g] = 0.43) and ADt (F = 26.29, eta2[g] = 0.35) and an increasing gradient was found in MDt (F = 49.30, eta2[g] = 0.50) and RDt (F = 34.16, eta2[g] = 0.41). The similar gradient changes were found in FA (F = 316.63, eta2[g] = 0.87), MD (F = 374.16, eta2[g] = 0.88) and RD (F = 382.27, eta2[g] = 0.89), while an opposite change was found in AD (F = 306.86, eta2[g] = 0.86).

### Correlations between diffusion metrics in subregions with different levels of FW

**Fig. 6:**
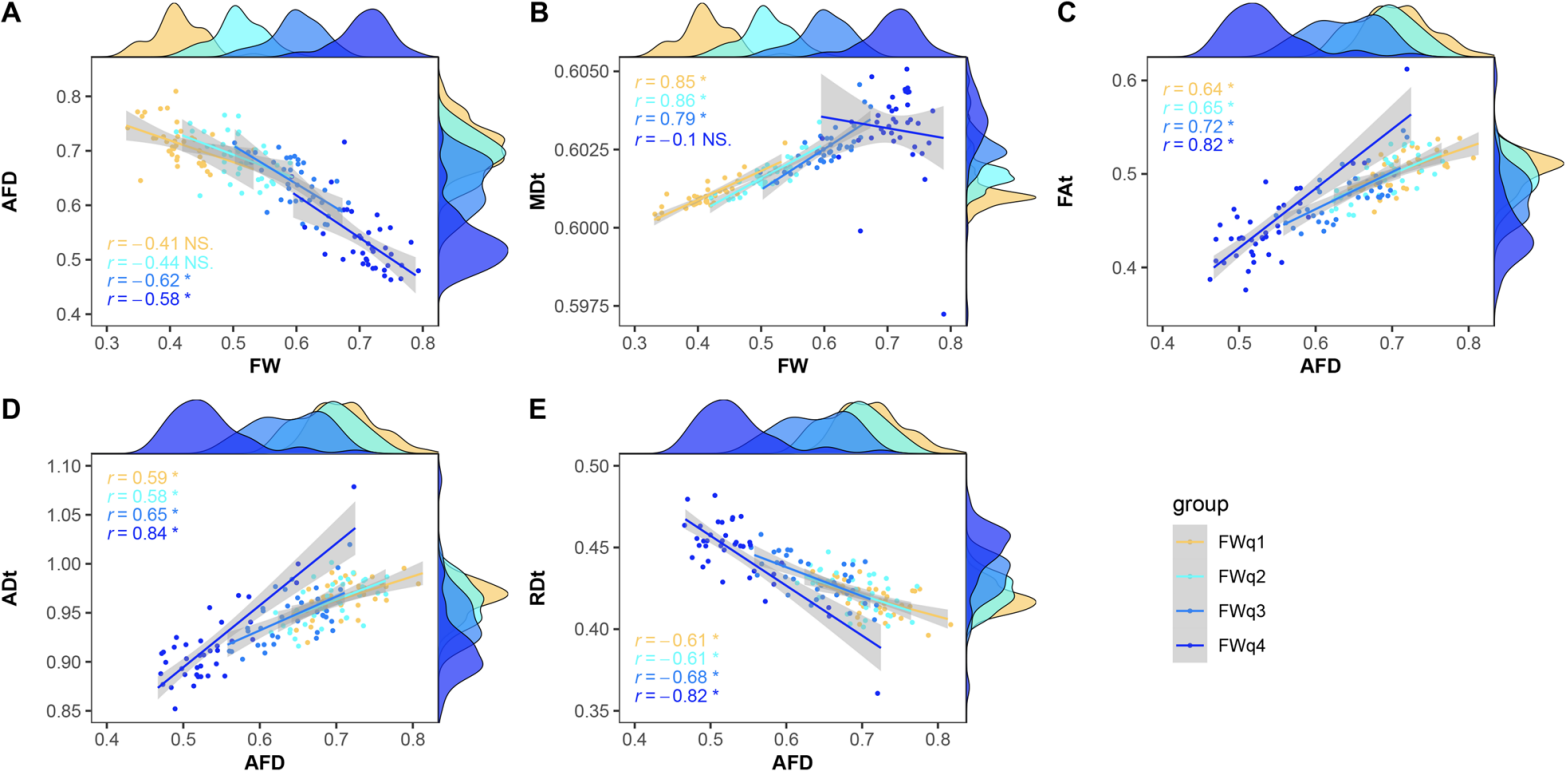
Correlations between FW, AFD with FW-corrected DTI metrics in each subregion of WMHs. **A** FW correlated with AFD in FWq3 and FWq4 (both *p* < 0.001), and marginally correlated with AFD in FWq2 (*p* = 0.006). **B** FW correlated with MDt in FWq1, FWq2 and FWq3 (all *p* < 0.001). **C**–**E** AFD correlated with FAt, ADt and RDt in each subregion of WMHs (all *p* < 0.001). The MDt, ADt and RDt values are in units of × 10^−3^ mm^2^/s. *Indicates Bonferroni corrected *p* < 0.05; NS. indicates not significant. *FW* free water, *AFD* apparent fiber density, *DTI* diffusion tensor imaging, *WMHs* white matter hyperintensities, *MDt* tissue compartment mean diffusivity, *FAt* tissue compartment fractional anisotropy; *ADt* tissue compartment axial diffusivity, *RDt* tissue compartment radial diffusivity, *FWq1-4* four subregions of WMHs split by FW quartiles

Increased FW was correlated with a decreased AFD in FWq3 and FWq4, and correlated with a higher MDt in FWq1, FWq2 and FWq3. Decreased AFD was correlated with a lower FAt, ADt and a higher RDt in each subregion of WMHs, and the correlation coefficient becomes stronger with the increased level of FW. The scatter plots of the correlations in each subregion of WMHs are shown in Fig. 6. After adjusting age, gender and corrected WMHs volumes, all the significant correlations were slightly improved (Additional file 1: Table S2).

### Relationships with duration of disease

No significant correlations were found between disease duration with FW and AFD in WMHs (r = 0.137, *p* = 0.431 and r = − 0.271, *p* = 0.115). In four subregions of WMHs, only marginal correlations were found between AFD and duration of disease in FWq3 and FWq4 (r = − 0.305, *p* = 0.075 and r = − 0.285, *p* = 0.097).

### Relationships with lacunes and microbleeds

**Fig. 7:**
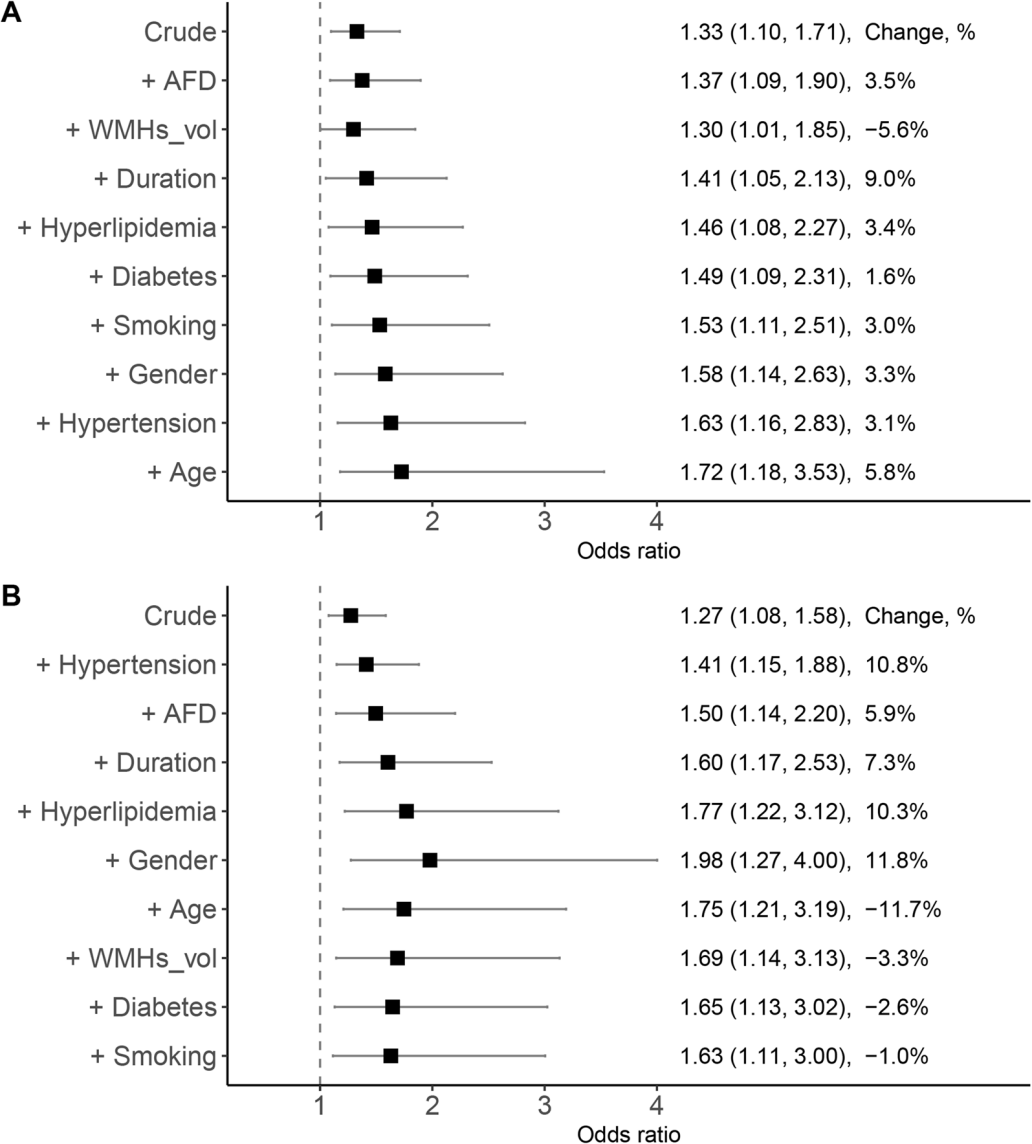
Confounding effects for each variable related with lacunes and microbleeds using CIE method. CIE calculates the changes in effect estimates when each variable is added to the model sequentially in a step-wise fashion. The odds ratio for free water without adjustment was presented in the row marked as Crude. **A** No variable meets the CIE criterion (a threshold of 10%) for lacunes. **B** Only hypertension meets the CIE criterion (a threshold of 10%) for microbleeds. *CIE* Change-in-estimate, *AFD* apparent fiber density, *WMHs_vol* corrected volume of white matter hyperintensities by intracranial volume

The interrater agreements for evaluating the presence of lacunes and CMBs were excellent (kappa = 0.87, 95% CI = 0.69–1.0; kappa = 0.95, 95% CI = 0.85–1.0; respectively). Table 2 showed the relationships between FW with lacunes and microbleeds. The changes in effect estimates after each variable added to the models are shown in Fig. 7. Increased FW independently correlated with the presence of lacunes (OR = 1.33, 95% CI = 1.10–1.71, *p* = 0.01). Increased FW also independently correlated with the presence of microbleeds (OR = 1.27, 95% CI = 1.08–1.58, *p* = 0.01 and OR = 1.41, 95% CI = 1.15–1.88, *p* = 0.005 after adjusting the confounder of hypertension).

**Table 2:** The relationships between free water with lacunes and microbleeds using logistics regression model

	Lacunes			Microbleeds		
	OR (95% CI)	*p* value	R^2^	OR (95% CI)	*p* value	R^2^
Model 1	1.33 (1.10–1.71)	0.010	0.31	1.27 (1.08–1.58)	0.010	0.27
Model 2	–	–	–	1.41 (1.15–1.88)	0.005	0.45

Model 1: crude, no adjustment; Model 2: adjusting for variables meeting the criteria of change-in-estimate approach (10% cutoff)

## Discussion

This study applied two advanced diffusion imaging methods to obtain deeper insight into the pathologic underpinnings of WMHs in CADASIL. In line with our hypothesis, we found an association between increased level of ECF and fiber microstructural changes in WMHs. Furthermore, we found that ECF was independently associated with both lacunes and microbleeds.

The etiology of WMHs involves complex mechanisms such as ischemia [27], blood–brain barrier (BBB) leakage [28], venous collagenosis [29] and impaired perivascular drainage [11]. Most of these mechanisms could lead to interstitial fluid (ISF) accumulation in extracellular spaces and the role of ECF in WMHs has been consistently reported in recent studies. For example, increased aortic stiffness could promote an increase in extracellular water content in WM, eventually leading to the development of WMHs [30]. While the strong relationship between collagenosis of the deep medullary veins and periventricular WMHs has been confirmed in radiological-pathological correlation studies [29, 31, 32], our recent study further suggests that their association is mediated by increased ECF [33]. Moreover, enlarged PVS is hypothesized to represent impaired drainage of ISF from the brain [34] and the fact that WMHs may grow from dilated PVS also suggests significant contribution of ECF accumulation [35].

To understand the distribution of different levels of ECF within WMHs in our cohort of CADASIL patients, we classified WMHs into four subregions based on the quartiles of FW and mapped the lesion probability of WMHs at each voxel for each quartile of FW. We found that the distribution of ECF in WMHs varied with its levels. Low-level ECF locates mainly at the deep WMHs in frontal and parietal lobes with a diffusely distributed pattern, whereas high-level ECF preferred to locate at the periventricular WMHs. Traditionally, the regional localization of WMHs such as periventricular and deep WMHs would be influenced by different features of vascular anatomy [36]. However, this theory seems difficult to explain the regional localization of ECF because a high level of ECF is also found in the deep WMHs of temporal lobe (i.e., anterior temporal lobe) which is a characteristic region in CADASIL. Our finding of increased ECF in WMHs of the anterior temporal lobe is consistent with a previous neuropathological study showing that increased ECF is related with enlarged PVS [7] which is an imaging marker of impaired ISF drainage [34]. Therefore, it can be speculated that regional variation of increased ECF in WMHs may reflect the degree of reduced capacity for ISF drainage from the WM.

Under normal conditions, the drainage of ISF and soluble metabolites from brain tissues by the perivascular pathways is necessary to maintain homeostasis in the brain [37]. A pathological hallmark of CADASIL is the early depositions of granular osmiophilic material (GOM) adjacent to basement membranes of arteries, arterioles, and capillaries [38]. It has been suggested that GOM plays a key role in hindering perivascular drainage [38]. Furthermore, the degeneration of vascular smooth muscle cells (VSMCs) in CADASIL reduces vascular tone and amplitude of pulsations for the propagation of perivascular lymphatic drainage, resulting in further accumulation of ISF [39]. The accumulation of ECF could cause a build-up of harmful substances such as plasma proteins from a leaky BBB [10], which are toxic to surrounding WM microstructures including myelin and axon. From a pathophysiological perspective, increased ECF possibly occurs first, followed by demyelination and axonal damage within WMHs.

The findings from our study may provide support for the above-mentioned theory. First, microstructural changes (significant correlations between FW and AFD, significant decrease in FAt and ADt, significant increase in MDt and RDt) only existed in WMHs subregions with high-level FW (FWq3 and FWq4), implying that WM microstructural damage starts to rise when ECF reaches above the median value of FW. Second, disease duration had a negative correlation (marginally significant) with AFD but not with FW in FWq3 and FWq4, suggesting that fiber disruption occurred at relatively late disease stages. Besides, in a recent study in a large cohort of older people of similar age, MD showed the best differentiation of WMHs from NAWM, suggesting that altered water mobility in the interstitial space may be an early feature of WM pathology [40]. Due to the cross-sectional nature of the current study, we could not rule out the possibility that WM microstructural degeneration results in the accumulation of ECF. However, if microstructural degeneration occurred before the event of ECF in WM, the atrophy of WM should be observed, like Wallerian degeneration in ischemic stroke [41]. Conversely, a neuroimaging study showed that patients with CADASIL had larger WM volume than controls [42], whereas WM atrophy was rarely reported in CADASIL.

Our findings of microstructural changes (decreased AFD, FAt, ADt and increased MDt, RDt) in WMHs were consistent with previous pathological findings [43, 44]. Combined with findings of ECF distribution, microstructural damage in periventricular WMHs is more severe than that in deep WMHs.

The diffusion alterations in FAt and MDt have been described in a recent CADASIL study [8]. To better characterize the microstructural damage related with axonal degeneration and demyelination, we used additional FW-corrected DTI metrics including ADt and RDt. Interestingly, ADt showed an opposite change in WMHs in contrast to AD, whereas other metrics showed the same direction in changes as the conventional DTI metrics. AD may increase with increased extracellular water content and reduced density of WM fibers, which allows faster water molecule movement parallel to axons [45]. This explanation is supported by our findings of increased FW and decreased AFD. The decreased ADt with increased AD has also been reported in white matter regions heavily affected by age [46]. Although our observation of decreased ADt and increased RDt in WMHs possibly respectively represent the pathological features of axonal degeneration and demyelination [47], interpretation of them remains to be challenged by other pathological changes in WMHs like gliosis, neuroinflammation and vascular alterations.

Apart from widespread WMHs, other imaging markers including lacunes and microbleeds appearing later on brain MRI, are also related with cognitive impairments in patients with CADASIL [48, 49]. We further found that increased ECF in WMHs was independently related to both lacunes and microbleeds, which was similar to our findings from sporadic CSVD [50]. As previously mentioned, the vessel wall structure is already weak due to VSMCs degeneration in CADASIL. On this basis, the increased ECF could further aggravate the weakness of the vessel wall with compromised autoregulation of cerebral blood flow, thus resulting in insufficient perfusion and vascular rupture. Therefore, WMHs, lacunes and microbleeds may share a common pathogenesis. It should be noted that lacunes and microbleeds can in turn contribute to the increased ECF. ISF clearance in the brain is driven by arterial blood flow, while decreased perfusion caused by lacunes and microbleeds can reduce the efficiency of solute clearance by this pathway [51, 52].

Several limitations should be considered. First, this is a cross-section study and thus, the relationships between diffusion metrics (e.g., FW and AFD) should be interpreted logically and cautiously. Future follow-up studies can be conducted to prove the effects of ECF on microstructural changes in WM. Second, analysis of PVS could offer complementary evidence about the impaired drainage of ECF in the brain, such as in the anterior temporal lobe. Unfortunately, due to lack of T2-weighted images, we did not perform the analysis of PVS. Third, there is currently limited histopathological evidence to specifically validate diffusion MRI metrics. Although measuring water distribution in postmortem tissue is challenging, future studies using novel methods to make direct comparisons between new diffusion metrics and pathology will be significant to improve the specificity of diffusion markers in CADASIL. Last, the sample size is relatively small. Nevertheless, the effect size in MANOVA test reaches medium to high, indicating the reliability of our findings.

## Conclusions

In summary, we demonstrated in CADASIL that increased ECF was associated with WM degeneration and the occurrence of lacunes and microbleeds, providing important new insights into the role of ECF in CADASIL pathology. Improving ECF clearance might become a therapeutic strategy in future.

## Supplementary Information

**Additional file 1.** Additional methods, tables and figure.

## Data Availability

The datasets generated and/or analysed during the current study are not publicly available due to ongoing analysis for future publication, but are available from the corresponding author on reasonable request.

## Abbreviations

AD: Axial diffusivity
ADt: Tissue compartment AD
AFD: Apparent fiber density
ANTs: Advanced Normalization Tools
BBB: Blood–brain barrier
CADASIL: Cerebral autosomal-dominant arteriopathy with subcortical infarcts and leukoencephalopathy
CSD: Constrained spherical deconvolution
CSF: Cerebrospinal fluid
CSVD: Cerebral small vessel disease
DTI: Diffusion tensor imaging
ECF: Extracellular fluid
EPI: Echo-planar imaging
FA: Fractional anisotropy
FAt: Tissue compartment FA
FLAIR: Fluid-attenuated inversion recovery
FODs: Fiber orientation distributions
FSL: FMRIB Software Library
FW: Free water
GM: Gray matter
GOM: Granular osmiophilic material
ICV: Intracranial volumes
ISF: Interstitial fluid
LPM: Lesion probability map
MANOVA: One-way multivariate analysis of variance
MD: Mean diffusivity
MDt: Tissue compartment MD
NAWM: Normal appearing white matter
OR: Odds ratio
PVS: Perivascular spaces
RD: Radial diffusivity
RDt: Tissue compartment RD
SS3T-CSD: Single-shell 3-tissue CSD
WM: White matter
WMHs: White matter hyperintensities
VSMCs: Vascular smooth muscle cells
