# Supplementary material for: Increased extracellular fluid is associated with white matter fiber degeneration in CADASIL: in vivo evidence from diffusion magnetic resonance imaging
Source: Fluids Barriers CNS. 2021 Jun 30;18:29. doi: 10.1186/s12987-021-00264-1 (PMC8247253; doi:10.1186/s12987-021-00264-1)
Supplement: Supplementary file 1 — Additional file 1. Additional methods, tables and figure. [file 12987_2021_264_MOESM1_ESM.docx]

**SUPPLEMENTARY MATERIAL**

**Increased extracellular fluid is associated with white matter fiber degeneration in CADASIL: in vivo evidence from diffusion magnetic resonance imaging**

**Supplementary Methods:**

MRI protocol: (a) Diffusion weighted imaging (DWI) data was acquired using echo-planar imaging (EPI) with the following parameters: TR/TE = 8000/80.8 ms, flip angle = 90°, slice thickness = 2 mm, matrix size = 128 × 128, FOV = 25.6 cm, 30 non-collinear gradient directions at b = 1000 s/mm2 and 5 volumes of b = 0 s/mm2, 2 mm isotropic voxels. (b) T2 fluid-attenuated inversion recovery (FLAIR) images were obtained with the following parameters: TR/TE = 8400/152 ms, TI = 2100 ms, flip angle = 90°, slice thickness = 4 mm, matrix size = 256 × 256, FOV = 24 cm. (c) 3D structural T1-weighted imaging (T1WI) was collected using a fast spoiled gradient recalled sequence with the following parameters: TR/TE = 7.3/3.0 ms, TI = 450 ms, flip angle = 8°, slice thickness = 1 mm, matrix = 250 × 250, FOV = 25 cm, 1 mm isotropic voxels. (d) Susceptibility weighted imaging (SWI) using a 3D high-resolution flow-compensated multi-echo sequence was performed with TR/TE = 34/4 to 30ms with 8 echoes, flip angle = 20°, slice thickness = 2 mm, matrix = 416 × 384 interpolated into 512 × 512, FOV = 24 cm, voxel size = 0.468 mm × 0.468 mm × 2 mm.

**Supplementary Figure S1. Comparisons of diffusion metrics between FWq1 and NAWM**

**
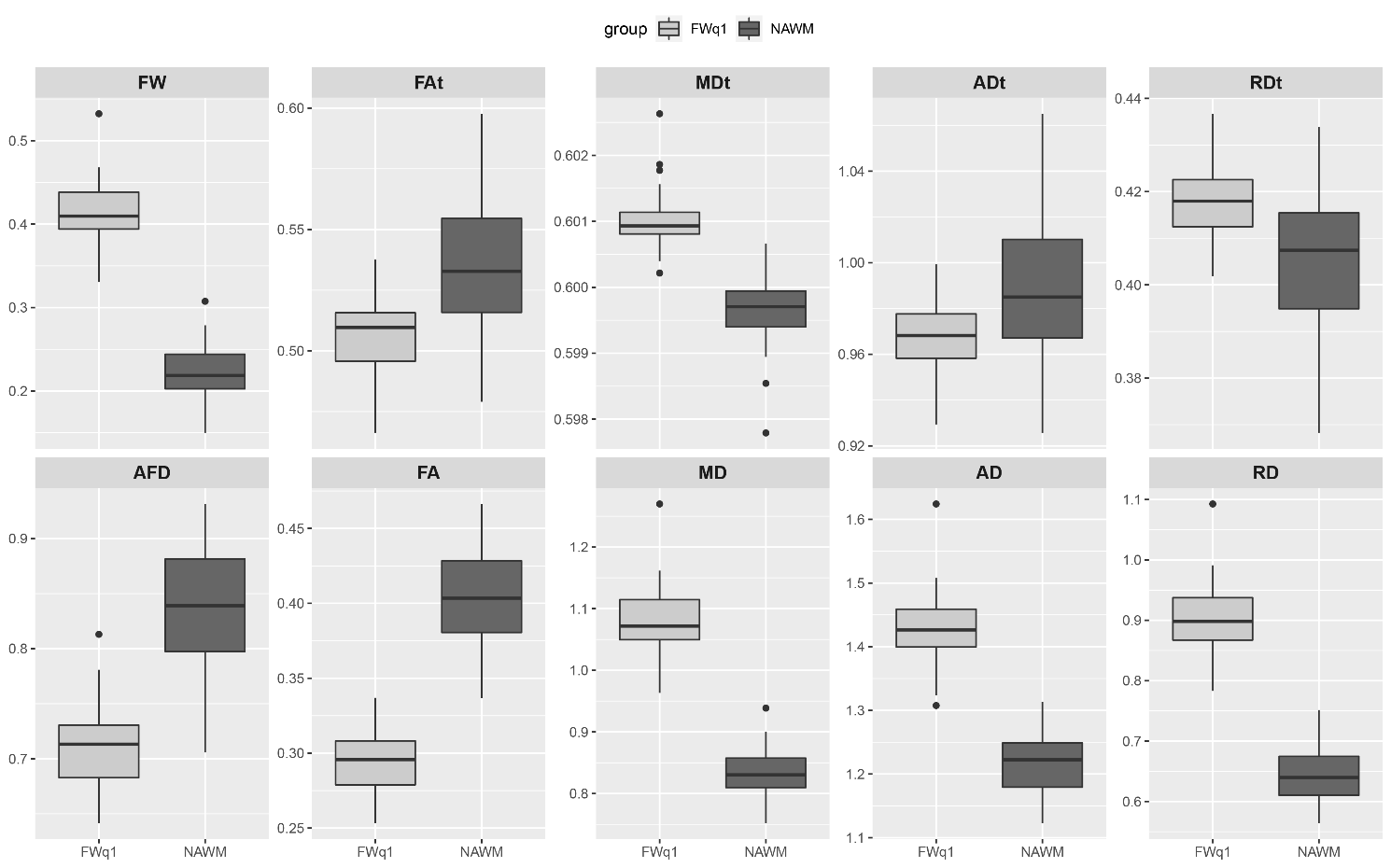
**

Significant differences were found in all diffusion metrics including FW, AFD, FAt, MDt, ADt, RDt, FA, MD, AD and RD (all p < 0.001) by paired-t test. The MDt, ADt, RDt, MD, AD and RD values are in units of ×10^−3^ mm^2^/s.

Note: FWq1 = WMH subregion divided by the first quartile of FW; WMHs = white matter hyperintensities; NAWM = normal appearing white matter; FW = free water; FAt = tissue compartment FA; MDt = tissue compartment MD; ADt = tissue compartment AD; RDt = tissue compartment RD; AFD = apparent fiber density; FA = fractional anisotropy; MD = mean diffusivity; AD = axial diffusivity; RD = radial diffusivity.

**Supplementary Table S1. Correlations between FW and AFD with FW-corrected DTI metrics in WMHs**

| Metrics | FW | | | | AFD | | | |
| --- | --- | --- | --- | --- | --- | --- | --- | --- |
|  | uncorrected | | corrected^#^ | | uncorrected | | corrected^#^ | |
|  | r | p value | r | p value | r | p value | r | p value |
| FW | - | - | - | - | **-0.594** | **<0.0001** | **-0.626** | **<0.0001** |
| AFD | **-0.594** | **<0.0001** | **-0.626** | **<0.0001** | - | - | - | - |
| FAt | -0.112 | 0.503 | -0.140 | 0.423 | **0.632** | **<0.0001** | **0.653** | **<0.0001** |
| MDt | **0.784** | **<0.0001** | **0.790** | **<0.0001** | -0.217 | 0.190 | -0.196 | 0.259 |
| ADt | -0.098 | 0.556 | -0.115 | 0.510 | **0.591** | **<0.0001** | **0.607** | **<0.0001** |
| RDt | 0.168 | 0.313 | 0.183 | 0.293 | **-0.623** | **<0.0001** | **-0.637** | **<0.0001** |

# corrected by age, gender and WMHs volumes (corrected by ICV).

Note: FW = free water; AFD = apparent fiber density; DTI = diffusion tensor imaging; FAt = tissue compartment fractional anisotropy; MDt = tissue compartment mean diffusivity; ADt = tissue compartment axial diffusivity; RDt = tissue compartment radial diffusivity; WMHs = white matter hyperintensities; ICV = intracranial volumes.

**Supplementary Table S2. Correlations between FW and AFD with FW-corrected DTI metrics in each subregion of WMHs**

| Metrics |  | FW | | | | AFD | | | |
| --- | --- | --- | --- | --- | --- | --- | --- | --- | --- |
|  |  | uncorrected | | corrected^#^ | | uncorrected | | corrected^#^ | |
|  |  | r | p value | r | p value | r | p value | r | p value |
| FW | FWq1 | - | - | - | - | -0.407 | <0.011* | -0.428 | <0.010* |
|  | FWq2 | - | - | - | - | -0.437 | <0.006* | -0.488 | <0.003* |
|  | FWq3 | - | - | - | - | **-0.617** | **<0.0001**** | **-0.665** | **<0.0001**** |
|  | FWq4 | - | - | - | - | **-0.578** | **<0.0001**** | **-0.596** | **<0.0001**** |
| AFD | FWq1 | -0.407 | <0.011* | -0.428 | <0.010* | - | - | - | - |
|  | FWq2 | -0.437 | <0.006* | -0.488 | <0.003* | - | - | - | - |
|  | FWq3 | **-0.617** | **<0.0001**** | **-0.665** | **<0.0001**** | - | - | - | - |
|  | FWq4 | **-0.578** | **<0.0001**** | **-0.596** | **<0.0001**** | - | - | - | - |
| FAt | FWq1 | -0.070 | 0.675 | -0.069 | 0.692 | **0.638** | **<0.0001**** | **0.677** | **<0.0001**** |
|  | FWq2 | -0.007 | 0.967 | -0.021 | 0.905 | **0.647** | **<0.0001**** | **0.656** | **<0.0001**** |
|  | FWq3 | -0.203 | 0.222 | -0.296 | 0.084 | **0.723** | **<0.0001**** | **0.742** | **<0.0001**** |
|  | FWq4 | -0.163 | 0.328 | -0.283 | 0.100 | **0.823** | **<0.0001**** | **0.872** | **<0.0001**** |
| MDt | FWq1 | **0.847** | **<0.0001**** | **0.854** | **<0.0001**** | -0.083 | 0.620 | -0.104 | 0.553 |
|  | FWq2 | **0.855** | **<0.0001**** | **0.855** | **<0.0001**** | -0.093 | 0.579 | -0.118 | 0.498 |
|  | FWq3 | **0.793** | **<0.0001**** | **0.778** | **<0.0001**** | -0.204 | 0.220 | -0.221 | 0.202 |
|  | FWq4 | -0.104 | 0.535 | 0.109 | 0.532 | 0.275 | 0.095 | 0.255 | 0.140 |
| ADt | FWq1 | -0.026 | 0.876 | -0.025 | 0.889 | **0.592** | **<0.0001**** | **0.631** | **<0.0001**** |
|  | FWq2 | 0.039 | 0.818 | 0.030 | 0.865 | **0.580** | **<0.0001**** | **0.586** | **<0.0001**** |
|  | FWq3 | -0.171 | 0.305 | -0.240 | 0.165 | **0.649** | **<0.0001**** | **0.661** | **<0.0001**** |
|  | FWq4 | -0.228 | 0.169 | -0.323 | 0.058 | **0.841** | **<0.0001**** | **0.874** | **<0.0001**** |
| RDt | FWq1 | 0.096 | 0.566 | 0.095 | 0.586 | **-0.612** | **<0.0001**** | **-0.654** | **<0.0001**** |
|  | FWq2 | 0.026 | 0.875 | 0.035 | 0.842 | **-0.606** | **<0.0001**** | **-0.614** | **<0.0001**** |
|  | FWq3 | 0.241 | 0.145 | 0.307 | 0.073 | **-0.683** | **<0.0001**** | **-0.693** | **<0.0001**** |
|  | FWq4 | 0.220 | 0.185 | 0.339 | 0.046* | **-0.822** | **<0.0001**** | **-0.868** | **<0.0001**** |

# corrected by age, gender and WMHs subregional volumes (corrected by ICV); * p < 0.05 without Bonferroni correction; ** p < 0.00125 after Bonferroni correction (0.05/40 = 0.00125).

Note: FW = free water; AFD = apparent fiber density; DTI = diffusion tensor imaging; WMHs = white matter hyperintensities; FWq1 = WMH subregion with 1st quartile of free water values; FWq2 = WMH subregion with 2nd quartile of free water values; FWq3 = WMH subregion with 3rd quartile of free water values; FWq4 = WMH subregion with 4th quartile of free water values; FAt = tissue compartment fractional anisotropy; MDt = tissue compartment mean diffusivity; ADt = tissue compartment axial diffusivity; RDt = tissue compartment radial diffusivity; ICV = intracranial volumes.
